# Survey and Performance Analysis of Deep Learning Based Object Detection in Challenging Environments

**DOI:** 10.3390/s21155116

**Published:** 2021-07-28

**Authors:** Muhammad Ahmed, Khurram Azeem Hashmi, Alain Pagani, Marcus Liwicki, Didier Stricker, Muhammad Zeshan Afzal

**Affiliations:** 1 Department of Computer Science, Technical University of Kaiserslautern, 67663 Kaiserslautern, Germany; agk1248@gmail.com (M.A.); khurram_azeem.hashmi@dfki.de (K.A.H.); didier.stricker@dfki.de (D.S.); 2 Mindgrage, Technical University of Kaiserslautern, 67663 Kaiserslautern, Germany; 3 German Research Institute for Artificial Intelligence (DFKI), 67663 Kaiserslautern, Germany; alain.pagani@dfki.de; 4 Department of Computer Science, Luleå University of Technology, 971 87 Luleå, Sweden; marcus.liwicki@ltu.se

**Keywords:** object detection, challenging environments, low light, image enhancement, complex environments, state of the art, deep neural networks, computer vision, performance analysis

## Abstract

Recent progress in deep learning has led to accurate and efficient generic object detection networks. Training of highly reliable models depends on large datasets with highly textured and rich images. However, in real-world scenarios, the performance of the generic object detection system decreases when (i) occlusions hide the objects, (ii) objects are present in low-light images, or (iii) they are merged with background information. In this paper, we refer to all these situations as challenging environments. With the recent rapid development in generic object detection algorithms, notable progress has been observed in the field of deep learning-based object detection in challenging environments. However, there is no consolidated reference to cover the state of the art in this domain. To the best of our knowledge, this paper presents the first comprehensive overview, covering recent approaches that have tackled the problem of object detection in challenging environments. Furthermore, we present a quantitative and qualitative performance analysis of these approaches and discuss the currently available challenging datasets. Moreover, this paper investigates the performance of current state-of-the-art generic object detection algorithms by benchmarking results on the three well-known challenging datasets. Finally, we highlight several current shortcomings and outline future directions.

## 1. Introduction

Object detection is considered as one of the most important and elementary tasks in the field of computer vision. The problem of object detection deals with the identification and spatial localization of objects present in an image or a video [1]. The task of object detection covers a wide range of many other computer vision tasks, such as instance segmentation [2,3,4], visual question answering [5], image captioning [6,7], object tracking [8], activity recognition [9,10,11] and so on.

Object detection is a complex problem due to underlying high intra-class and low inter-class variance [12,13]. High intra-class variance is the consequence of different objects belonging to a single class, for instance, different poses of humans or humans wearing different clothes in an image. Low inter-class variance is the outcome of similar-looking objects belonging to different classes such as samples of class chair can easily be misclassified into the class bench and vice versa.

One of the earlier approaches for object detection algorithms relied on sliding windows, applying classification on each window to find objects [14,15,16]. Later, the sliding window concept was replaced with region proposals to narrow the search before applying classification [17,18,19,20,21]. The recent surge in deep learning has given rise to object detection systems along with other fields.

**Figure 1:**
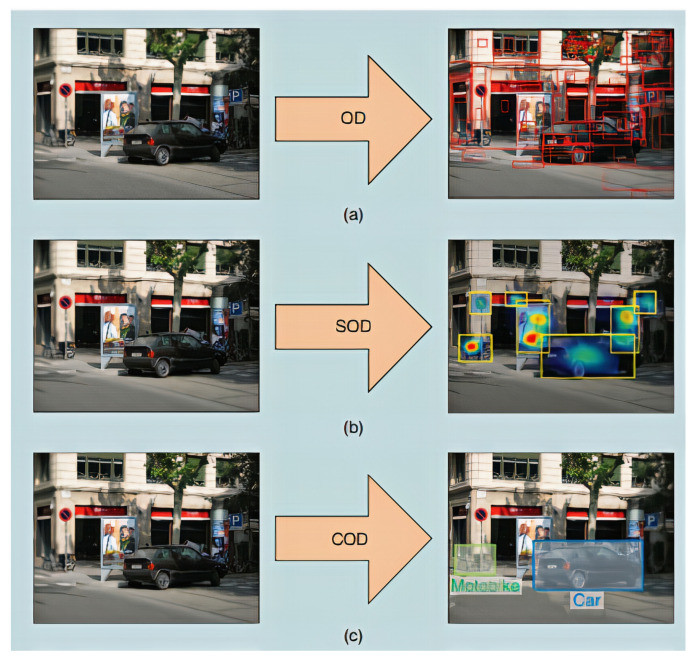
Visual demonstration of the possible three directions in the domain of object detection. Object detection (OD): objects are detected as illustrated in part (**a**), Salient Object Detection (SOD): human attention mechanism is applied to detect objects as presented in part (**b**), Category-specific object detection (COD): object detection along with the class prediction as depicted in part (**c**). Reprinted with permission from [30]. Copyright 2018 IEEE.

The prior published work in object detection can be further classified into three categories which are explained below. Figure 1 depicts the basic difference between them:

1. *Object Detection (OD)*: OD aims at detecting objects regardless of their class category [17,22]. OD algorithms [23,24,25,26] generally propose a large number of possible region proposals, from which, later on, the best possible candidates are selected according to certain criteria.
2. *Salient Object Detection (SOD)*: SOD algorithms use the human attention mechanism concept to highlight and detect the objects in a picture or video [27,28].
3. *Category-specific Object Detection (COD)*: COD aims at detecting multiple objects. Unlike OD and SOD, COD has to predict the category class and the location of the object in the image or video [16,29].

The deep learning-based object detection algorithms are categorized into two-stage object detectors and one-stage object detectors. Two-stage object detection architectures such as R-CNN [16], Fast R-CNN [31] and Faster R-CNN [23] segregate the task of object localization from the object classification task. They employ region proposal techniques to find possible regions where the likelihood of an object’s existence is maximum. Later segmentation output and better detection pooling [23] techniques were introduced with Mask R-CNN [25]. On the other hand, one-stage object detection algorithms first generate candidate regions, and then these regions are classified as object/no-object. For instance, one-stage detectors such as YOLO [24,32,33,34] and SSD [26] work with feature pyramid networks (FPNs) [35] as a backbone to detect objects at multiple scales in a single pass rather than first predicting regions and then classifying them.

**Figure 2:**
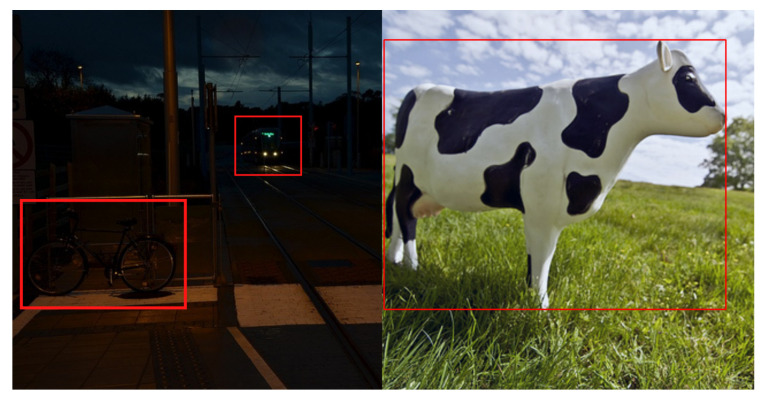
Graphical illustration of object detection in a challenging environment (left image), whereas object detection in a conventional environment is shown in the right image. There are two objects: bicycle and bus are present in the challenging left image while the cow is a targeted object in the right image.

Analogous to other domains of computer vision, the output of object detection is highly dependent on spatial features. Hence, the performance of the object detection system decreases when the occlusions hide the objects, objects have different sizes or are merged with background information. In real-time scenarios, it is frequent that the input images received by the object detection network are not rich with information, or they are captured under low-light conditions. This paper has referred to all these situations in a challenging environment. Figure 2 illustrates the problem of object detection in a challenging environment. This survey paper investigates the approaches that have worked on detecting objects in such environments by leveraging deep neural networks.

**Figure 3:**
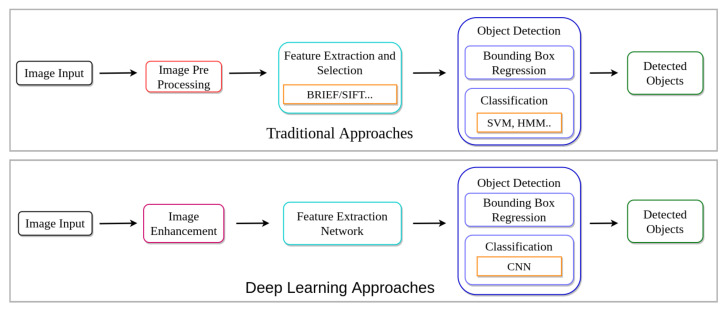
Pipeline comparison of traditional and deep learning approaches for object detection. In traditional approaches, generally, image enhancement is applied before feature extraction to improve object detection performance. Unlike traditional approaches, deep learning methods can find required features for detecting objects without relying on traditional rule-based methods.

Different pre-processing techniques have been applied to improve image quality to improve object detection performance in challenging environments over the years. Figure 3 illustrates the comparison between the flow of traditional approaches and deep learning-based methods. Traditional methods rely on improving image quality through image enhancement and manual feature selection methods [1,14,36,37]. Later, these techniques were replaced with Deep Neural Networks (DNNs) due to their robust and generalization capabilities.

### Contributions

**Figure 4:**
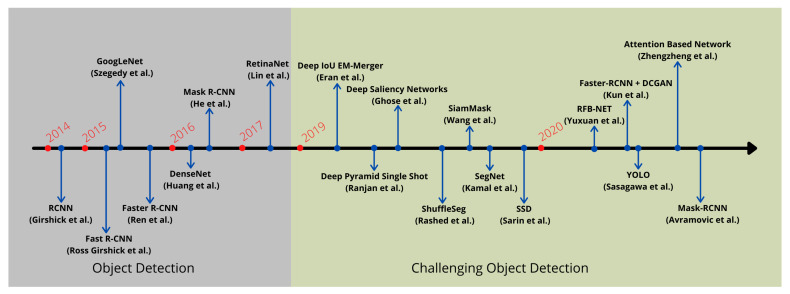
A timeline view of different deep learning approaches developed recently to improve object detection in challenging environments.

This survey paper focuses on the recent works that have tackled object detection in a challenging environment by employing deep neural networks. With the modern advancement of deep learning-based object detection approaches, a noticeable surge has been observed in the field of object detection under a complex environment, as illustrated in Figure 4.

To summarize, our contributions are as follows:

1. We present a unified framework that explains object detection in a challenging environment;
2. We give an overview of all the publicly available datasets that have been published to detect objects in challenging scenarios;
3. We summarize the advantages and limitations of the discussed methods in order to improve object detection in a challenging environment;
4. We benchmark current state-of-the-art generic object detection algorithms on the three well-known challenging datasets.

**Figure 5:**
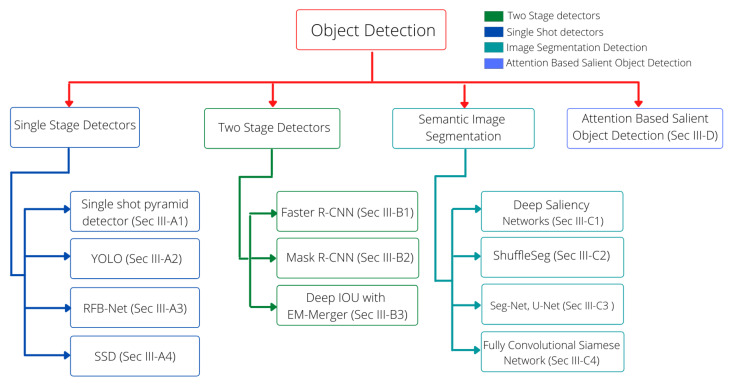
Organization of explained methodologies in this paper. The problem of object detection in challenging environments has been tackled by employing various deep learning concepts. In this paper, we categorize these methods according to the utilized architectures.

The remainder of the paper is organized as follows: Section 2 briefly discusses previous surveys conducted on object detection in challenging environments. Section 3 provides an exhaustive discussion on recently applied approaches to solving problems of object detection in difficult environments. Figure 5 explains the structural flow of mentioned methodologies. Section 4 provides details about publicly available datasets for challenging object detection tasks. Section 6 explains well-known evaluation metrics and provides performance of all the discussed approaches in Section 3. Section 7 highlights the current challenges and suggest future directions, and Section 8 concludes the paper.

## 2. Related Surveys on Object Detection

There are many surveys carried out on the topic of object detection [38,39,40,41]. This section covers some of the prior surveys.

Han et al. [30] organized the survey in which deep learning techniques for salient and category-specific object detection are reviewed. In 2019, Zou et al. [42] performed an extensive survey on object detection methods that have been proposed in the last 20 years. The authors discussed all the types of object detection algorithms proposed over the years and highlighted their improvements.

Another survey organized by Jiao et al. [43] discussed various deep learning-based methods for object detection. The proposed work provided a comprehensive overview of traditional and modern applications of object detection. Moreover, the authors discussed methods for building better and efficient object detection methods by exploiting existing architectures. Arnold et al. [44] surveyed 3D object detection methods for autonomous driving. The proposed work compared various 3D object detection-based approaches.

It is vital to mention that all of the prior surveys have focused on the general problem of object detection. Although these surveys explain how object detection has improved over the years, they do not cover the challenges and solutions to improve object detection performance in a challenging environment such as low light, occlusions, hidden objects, and so on. To the best of our knowledge, we provide the first survey that reviews the performance of deep learning-based approaches in the field of object detection in a challenging environment.

## 3. Methodologies

The process of detecting an object is divided into the following steps:

1. Find regions as object/no-object;
2. Classify the detected regions where objects exist.

**Figure 6:**
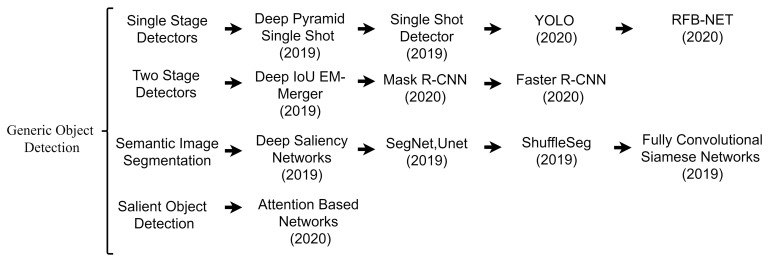
Different object detection algorithms approaches are categorized into four categories. One-stage detectors are fast but lack accuracy, whereas two-stage detectors are slow but accurate. Semantic image segmentation deals with pixel-level detection, and salient object detection deals with detecting objects by applying an attention mechanism.

**Figure 7:**
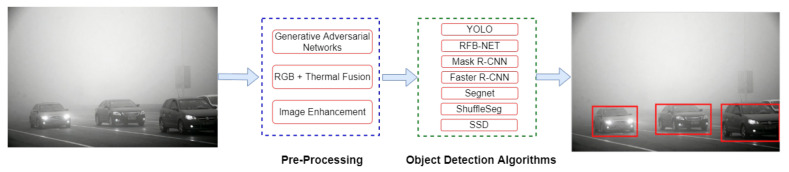
The generic mechanism of the proposed approaches that have tackled the problem of object detection in a challenging environment. Image-enhancement networks extract important features from the input images. Object detectors employ these features to retrieve final predictions.

This section discusses the various approaches that have tackled object detection problems under complex scenarios by employing deep neural networks. As explained earlier in Section 1, various object detection-based algorithms and a few semantic segmentation-based approaches have been exploited in the recent past to develop object detection systems under challenging situations. For convenience, we have categorized the methodologies according to the employed architectures. Figure 5 shows the structure of this section, whereas the categories of the explained methods are illustrated in Figure 6. Furthermore, Table 1 summarizes the main advantages and limitations of the mentioned approaches. Moreover, Figure 7 exhibits the generic mechanism of the recently proposed approaches in this domain.

### 3.1. One-Stage Detectors

**Figure 8:**
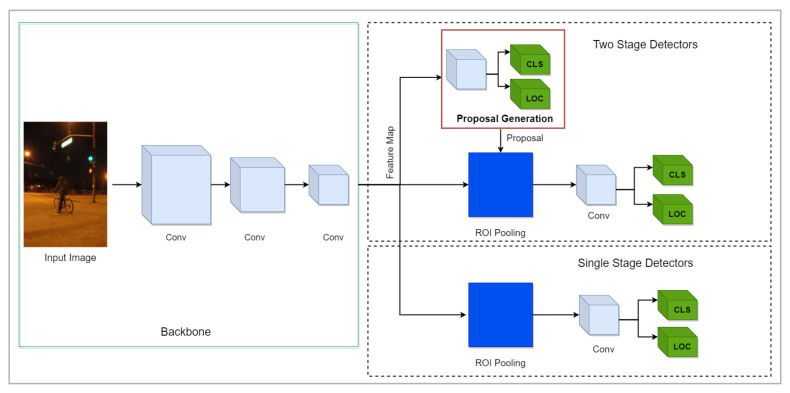
Graphical illustration of the object detection algorithms. The upper part of a figure exhibits two-stage detectors in which the backbone network extracts features that are passed to RPN to generate region proposals. These region proposals are refined to detect the objects in images. The lower part illustrates one-stage detectors in which objects are localized and classified in a single forward pass. The grey cubes in the backbone demonstrate the convolutional operation.

One-stage detectors predict the candidate regions without taking aid from region proposal networks. Compared to two-stage detectors [23,25], they are efficient and widely exploited in developing real-time object detection systems [45,46,47]. The architectural overview of one-stage detectors is depicted in Figure 8. Researchers have proposed approaches to detect objects in arduous environments by adopting one-stage detectors. This section further arranges the approaches according to the operated one-stage object detectors.

#### 3.1.1. YOLO

**Figure 9:**
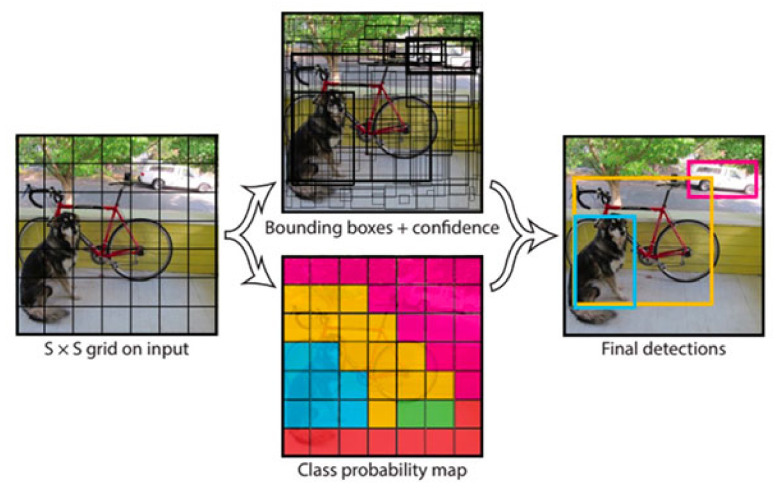
The explained architecture of YOLO [24]. YOLO sees the complete image at once and segments an image into S × S grids. Each cell predicts bounding boxes and a confidence score for every bounding box. The cell in which object is present at the center is liable to detect the object. Reprinted from [24].

You Only Look Once (YOLO) [24] has been widely applied to develop real-time object detection systems in various domains [45,46,47]. Unlike region-based convolutional neural networks, YOLO only takes a single look and divides the image into S×S grid of cells. For each object that exists on the image, one grid cell is supposed to be responsible for having that object at its center. Figure 9 illustrates the main idea of YOLO. This section covers the techniques that have exploited YOLO to detect objects in a complex environment.

By leveraging the power of transfer learning, Sasagawa et al. [48] proposed an approach to detect objects under low illumination. The work proposed combining two trained models from different but related domains through glue layers and a generative model. The authors have used the knowledge distillation technique to train the proposed approach. Initially, encoder–decoder network [49] is used to extract spatial features from the given image by using convolutional [50] and pooling layers [51]. The pooling layer is used to capture features at different frequencies of information. Once the encoder–decoder network learns the latent representation, it is propagated to the glue layer. The glue layer is composed of batch normalization [52], pooling, and concatenation. The authors empirically established that the concatenation of all latent features produces the optimal result. The glue layer uses RGB data generated by encoder $g_{2}$ using the knowledge distillation method. YOLO [24] is utilized to localize and identify objects. Since the proposed technique relies on pre-trained networks, the backbone network is pre-trained on MS-COCO [12] and See in the dark(SID) [53] datasets. By combining pre-trained models using glue layers, the proposed method could detect objects in scenes illuminated by less than 1 lux. Further, it is 2.4 times more sensitive than the original YOLO model [24]. Moreover, the use of the glue layer further reduces the computational resources.

Utilizing YOLO, Mate et al. [54] employed thermal images to improve object detection performance in challenging conditions such as bad weather, night-time, and densely packed areas. The authors argued that thermal images could easily show the difference between objects and the surrounding environment based on temperature values as compared to RGB images. This aids the process of detecting objects in difficult situations such as low-light scenarios, rainy or foggy weather. This work consists of two different experiments. The first network was fine-tuned on the thermal dataset UNIRI-TID [55], whereas the second model was trained from scratch on the same UNIRI-TID thermal dataset. The authors found that the model trained from scratch performed better than the fine-tuned model. The authors also reported that the model’s performance drops in the case of bird’s-eye view images.

#### 3.1.2. RFB-Net

**Figure 10:**
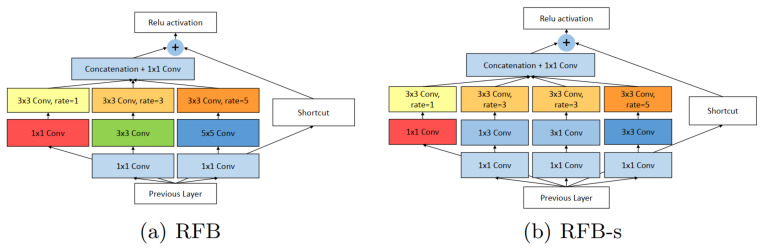
The explained architecture of RFB [56]. RFB is inspired by Receptive Fields (RFs) in human visual system. RFB considers the relationship between the size and eccentricity of receptive fields into account to enhance the features. (**a**) RFB is identical to Inception, responsible for simulating the RFs of multiple sizes, and (**b**) part reproduces the relation between the RF size and eccentricity in the human visual system by using smaller kernels. Reprinted from [56].

Currently, existing state-of-the-art detection algorithms use pre-trained backbones based on Feature Pyramid Network (FPN) to extract low-level and high-level features. Instead of relying on a pre-trained backbone, RFB-Net is inspired by the Receptive Fields (RFs) structure in the human visual system. RFB-Net considers the relationship between size and eccentricity of RFs to enhance features. Figure 10 illustrates the main building block of RFB-Net.

Existing state-of-the-art generic object detection algorithms do not perform well on images captured at low light because most low-level features are merged with the background. To tackle this problem, Yuxuan et al. [57] fused contextual information in the backbone to avoid loss of low-level contextual features. Lower spatial feature maps with higher spatial feature maps are fused to preserve low-level features, maximizing pre-trained channel information.

The second problem with images captured in low light is that when these images are passed through conventional hierarchical convolutions, the resulting edges and features are not very rich with information. Therefore context fusion is incorporated in the backbone part of the network. The aim is to compensate for the information loss during the lower to higher-level convolution process. Low-level feature maps of the network are selected and interpolated to the identical shape of its successive feature maps. The resulting feature map is rich in contextual information, combining high-level features and low-level features. The authors merged RFB-Net [56] as a base architecture with the proposed modifications.

#### 3.1.3. SSD

**Figure 11:**
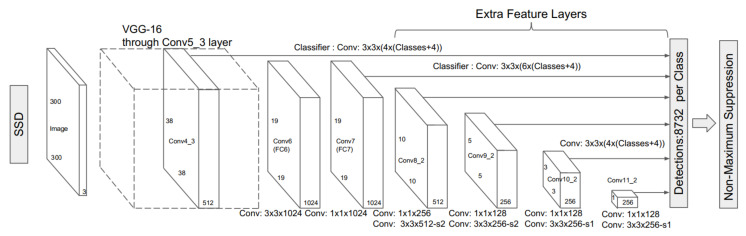
The explained architecture of SSD [26]. The architecture of SSD is based on the VGG-16 [70] as base network by replacing VGG fully connected layers with auxiliary convolutional layers to provide feature extraction at multiple scales. Later, the multibox detector [71] performs the task of object detection. Reprinted from [26].

SSD [26] is another extensively employed real-time object detection algorithm belonging to the family of one-stage detectors. SSD eliminates the region proposal network from the Faster R-CNN [23] to increase the network’s speed. SSD includes multiscale features and default boxes to compensate for the performance trade-off. The architecture of SSD is illustrated in Figure 11.

Sarin et al. [58] proposed a convolutional neural network-based human detection and SSD-based face detection system to improve the results of surveillance systems in challenging environments. The first human detection network is used to identify whether an input image contains a human. Once a human is detected, the method exploits SSD to localize the area of the face on a predicted image. Since the conventional method tends to over-fit on the provided dataset, the proposed work leverages dropout [50] and data augmentation techniques to avoid overfitting. For the enhancement of dark images, brightness normalization with the zero-center method was adopted. The model operated on AlexNet [50] and was modified for the problem at hand. Two datasets, the KAIST [59] and UFDD [60] datasets, were used to train human and face detection networks, respectively.

##### Deep Pyramid Single Shot Face Detector

For face detection in a harsh environment, Ranjan et al. [61] proposed a Deep Pyramid Single Shot face Detector (DPSSD). The authors argued that deep pyramidal architecture in SSD [26] can even detect small-scale faces in the images. In this work, the input image is passed through up-sampling layers to extract rich contextual features and then passed through pooling layers. Final detection boxes are generated from the up-sampling layers through the anchor box matching technique. The output prediction divides into two branches: regression and classification. Furthermore, to optimize network convergence, crystal loss is introduced for face verification and classification tasks. The proposed crystal loss minimizes the angular distance between similar subject pairs and maximizing the angular distance between different subject pairs. For the training purpose, the WIDER Face dataset [72] was employed. The proposed approach is extensively evaluated on unconstrained face detection datasets of IJB-A [73], IJB-B [74], and IJB-C [75].

### 3.2. Two-Stage Detectors

Along with one-stage detectors, two-stage detectors have been applied to tackle object detection in difficult situations. Figure 8 depicts the basic architecture of two-stage detectors.

#### 3.2.1. Faster R-CNN

**Figure 12:**
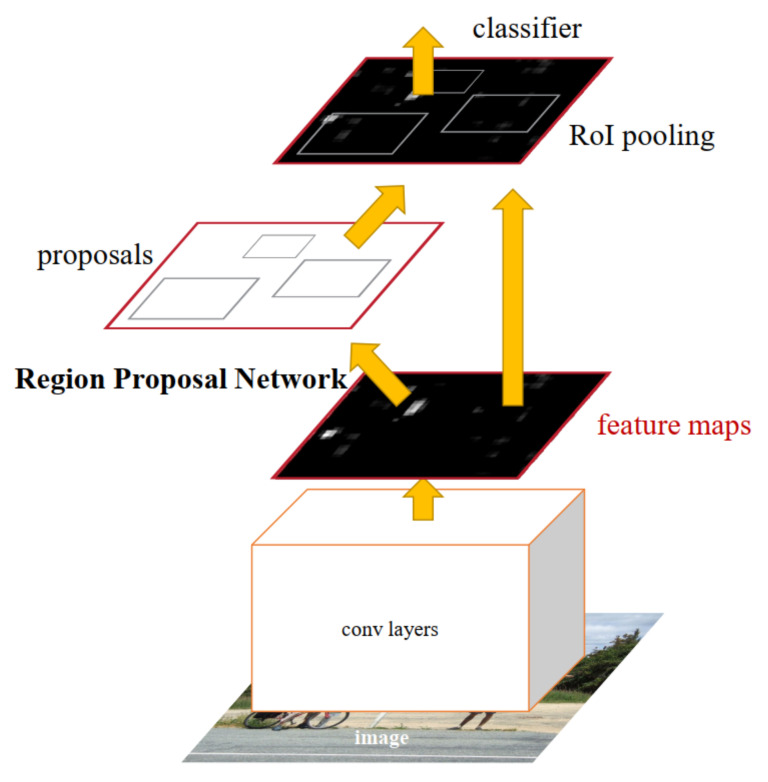
The explained architecture of Faster R-CNN [23]. The base network generates the feature maps from input images and propagates them to RPN and ROI pooling. The RPN generates region proposals which are refined by ROI pooling to perform regression and classification. Reprinted from [23].

Faster R-CNN [23] is one of the most widely exploited object detection networks [76,77]. It is the extension of Fast R-CNN [31] with Region Proposal Network (RPN). RPN aims to generate proposals with different sizes and aspect ratios. These proposals and the spatial features are propagated to the Fast R-CNN [31] module, which performs object detection. The basic architecture of Faster R-CNN [23] is depicted in Figure 12. This section discusses the methodologies that employ Faster R-CNN [23] as an object detection algorithm in their system of identifying objects under various difficult scenarios.

Generative Adversarial Networks (GANs) [78] have also been applied to enhance the visual quality of images captured under low light. Kun et al. [64] proposed the Deep Convolution Generative Adversarial Networks (DCGANs) [79] combined with Faster R-CNN [23] to solve the problem of object detection in low light. The first step is to use DCGAN to convert night-time scenes into daytime scenes by keeping the important aspects maintained between two input images. The second step is multiscale convolution feature fusion. This work applies up-sampling and down-sampling convolutions to fuse features extracted from starting layers.

The third contribution of this work is the proposed modification in the ROI pooling layer. According to the coordinate vector, the standard ROI pooling layer uses bilinear interpolation to obtain pixel value. Although conventional ROI pooling reduces parameters, the target object’s critical features are lost. Therefore, the authors employed ROI pooling of different sizes to capture more detailed information. Subsequently, the final ROI pooling output is passed to the classifier and regressor to obtain final predictions.

#### 3.2.2. Mask R-CNN

**Figure 13:**
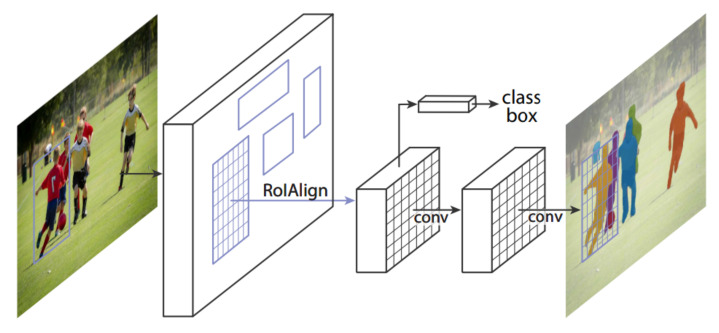
The explained architecture of Mask R-CNN [25]. The architecture of Mask R-CNN extends the architecture of Faster R-CNN [23] with two modifications: (1) replacing the RoI pooling with RoIAlign layer; (2) addition of segmentation branch along with regression and classification. Reprinted from [25].

In the family of region-based convolutional neural networks, Mask R-CNN [25] has been exploited by the state-of-the-art object detection and instance segmentation approaches [80,81]. Mask R-CNN [25] extends Faster R-CNN [23] with an addition of another branch that retrieves the mask for each detected object. The complete framework of Mask R-CNN is illustrated in Figure 13. This section covers the approaches that have tackled object detection under strenuous conditions by exploiting the capabilities of Mask R-CNN.

Another approach for detecting traffic signs is proposed by Avramovic et al. [62]. The authors discussed that a driver could only focus in front of him and beside him through side mirrors during driving. Hence, to detect traffic signs, the model should perceive an input image in the same way. Therefore, instead of applying object detection to the whole image, the proposed work focuses on a specific region where a traffic sign is likely to be seen. Selecting a limited amount of Regions Of Interests (ROIs) reduces the overall computation cost and propagates smaller images through the network efficiently. Furthermore, the original aspect ratio of traffic signs is preserved by using a small number of ROIs. They evaluated the approach equipped with Mask-RCNN [25] and YOLO [24] on the DFG traffic sign dataset [82].

#### 3.2.3. Deep IOU with EM-Merger

In a challenging environment, generic object detectors predict multiple bounding boxes for a single object. To address this issue, Eran et al. [63] proposed a Soft-IOU layer for estimating Jackkard distance as a quality detector between the predicted bounding box and the ground truth. The presented approach aims to minimize overlap regions between multiple detections. Once the network predicts the bounding boxes for the input image, the predictions are treated as a clustering problem. The method applies a custom EM-Merger layer to group similar predictions to form a single detection.

Although conventional non-max suppression can remove overlapping detection, the authors argued that non-max suppression does not provide a satisfactory result in densely packed situations. This work empirically established that it is better to use the EM-Merger layer to combine extra detections. Their proposed approach achieved better results than the state-of-the-art object detection-based algorithm on SKU-110K benchmark dataset [63].

### 3.3. Semantic Image Segmentation

**Figure 14:**
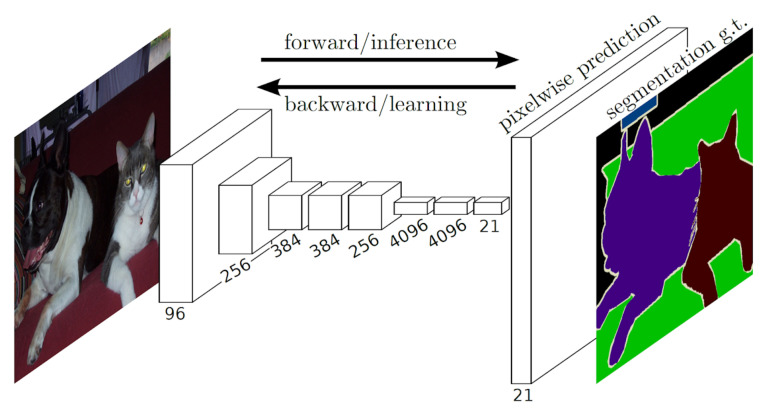
The explained architecture of Fully Convolutional Networks (FCNs). FCN first down-samples and then transforms the image back to the original spatial size using up-convolutions. Using dense predictions, it tries to predict a class label for each pixel in an image and segment the objects based on the classes of pixels. Reprinted from [84].

In addition to generic object detection algorithms, researchers have investigated the capabilities of Semantic Image Segmentation (SIS) [83] in identifying objects in arduous conditions. Instead of localizing an object in an image, SIS predicts a class label for each pixel in an image. This task is also termed a dense prediction. Once we acquired the labeled pixels, we segmented the objects by grouping the pixels belonging to the same class. The methods based on SIS mainly operate on Fully Convolutional Networks (FCNs) [84]. Unlike other deep neural networks, FCNs consist of only convolutional layers and no fully connected layers. Therefore, FCNs can work with images of various sizes. A visual illustration of FCNs are exhibited in Figure 14. This section addresses some of the methods that operate on the concept of SIS to deal with object detection in perplexing scenarios.

#### 3.3.1. Deep Saliency Networks

In 2019, Ghose et al. [65] proposed the combination of saliency maps with thermal images to detect pedestrians in poor lighting conditions. This work suggested that instead of relying on thermal and RGB images, the addition of saliency map information to thermal images facilitates the network to identify the objects. At first, the method augments the thermal images by replacing one duplicate channel with a corresponding saliency map. This combination helps illuminate salient parts of the image while preserving textural information. To extract saliency maps, the authors experimented with two state-of-the-art deep saliency networks (PiCA-Net [85] and R3Net [86]).

PiCA-Net generates an attention map for each pixel in an image and employs Long Short Term Memory (LSTM) [87] to scan the image and obtain global context. U-Net [88] is then applied to detect salient objects from the generated attention maps. R3Net [86] uses residual refinement block to learn residuals between the ground truth and saliency maps. To assess the performance of the proposed method, the authors exploited KAIST multispectral pedestrian dataset [59].

#### 3.3.2. ShuffleSeg

Following the concept of applying image segmentation [65], Rashed et al. [66] proposed an FCN-based architecture for moving object detection by concatenating spatial information with LiDAR sensors. The presented work employed three encoders to handle RGB, rgbFlow, and lidarFlow information separately. The whole network follows encoder–decoder architecture. The encoder extracts the latent features while the decoder up-samples them to generate the final image. To analyze the capabilities of the proposed approach, the authors used KITTI dataset [89] and a custom-made Dark-KITTI dataset to simulate a low-light environment. The proposed method achieved a 4.25% improvement on standard KITTI and 10.1% relative improvement on the Dark-KITTI dataset.

#### 3.3.3. SegNet, U-Net

Kamal et al. [67] integrated two different network architectures based on FCNs [84] to localize and classify traffic signs. This approach operated on a combination of SegNet [90] and U-Net [88] to detect segment signs and a VGG-16 [70] network for the classification.

Initially, the method extracts patches from all the corners of the input images. The models (SegNet and U-Net) train on these patches to predict the segmented traffic signs. Then, predictions of the four patches are merged to make an output mask for the original image. VGG-16 [70] categorizes the detected sign into 14 various classes. To increase the detection of small traffic signs, the authors used the L1 constraint term to modify Tversky Loss [91] instead of the conventional intersection over union loss. To appraise the system, the author used the CURE-TSD dataset [92] and achieved a precision of 94.60% and recall of 80.21% beating previous state-of-the-art results.

#### 3.3.4. Fully Convolutional Siamese Networks

Utilizing fully convolutional Siamese networks [93], Wang et al. [68] proposed *SiamMask*, a modified network with an addition of a binary segmentation task. This work explained the importance of producing per-frame binary segmentation masks instead of relying on low-level object representations extracted from existing spatial extraction networks [70].

As siamese networks [93] can conveniently encode the required information to produce a pixel-wise binary mask, the method adds a different branch and loss to the existing architecture. The first modification generates a binary mask by adding a simple two-layer neural network with learnable parameters during training. Secondly, the authors proposed binary logistic regression loss for the new branch.

Unlike Mask R-CNN [25] and other segmentation architectures that rely on maintaining spatial information throughout the network, the proposed approach generates masks starting from a flattened representation of the object. For evaluation, two networks, SiamFC [93] and SiamRPN [94], were modified to perform experiments on the VOT-2018 dataset [95]. SiamMask was able to perform better than prior existing algorithms.

### 3.4. Attention-Based Salient Object Detection

Employing salient object detection Zhengzheng et al. [69] fused RGB images with thermal images to extract multilevel features to detect objects in adverse conditions. The proposed work used a two-stream convolution neural network architecture to generate RGB and thermal image features.

The proposed work exploited multiple attention mechanisms to extract weighted features from both RGB and thermal input images. The extracted weighted features are then fused to form single input for later convolutions. These feature maps are passed through lower-level to high-level convolution blocks in a standard forward pass fashion. Although high-level features are vital, mid-level features are essential to refine object details. Therefore, this work added Pyramid Pooling Module and a feature aggregation Module to sharpen the object details and facilitate localizing the object.

To remove noise from features, the approach adds the Convolutional Block Attention Module(CBAM) [96] to apply channel-wise and spatial-wise attention. Later, average pooling aggregates the spatial information from feature maps while max-pooling generates two different spatial context information. Subsequently, the approach merged the spatial context features and applied the attention mechanism with the sigmoid. During the training, edge loss and cross-entropy loss are added together. The proposed method was tested on the VT5000 dataset [69].

## 4. Datasets

The performance of deep neural network-based approaches directly relates to the size of datasets. In any area of research, standard datasets are essential to benchmark the performance and draw fair comparisons between various proposed approaches. Several challenging large-scale datasets have been introduced that consist of images or videos captured in a harsh environment. In this section, we take a look at publicly available challenging datasets. Moreover, Table 2 summarizes the essential information of the explained datasets.

### 4.1. ExDARK

**Figure 15:**
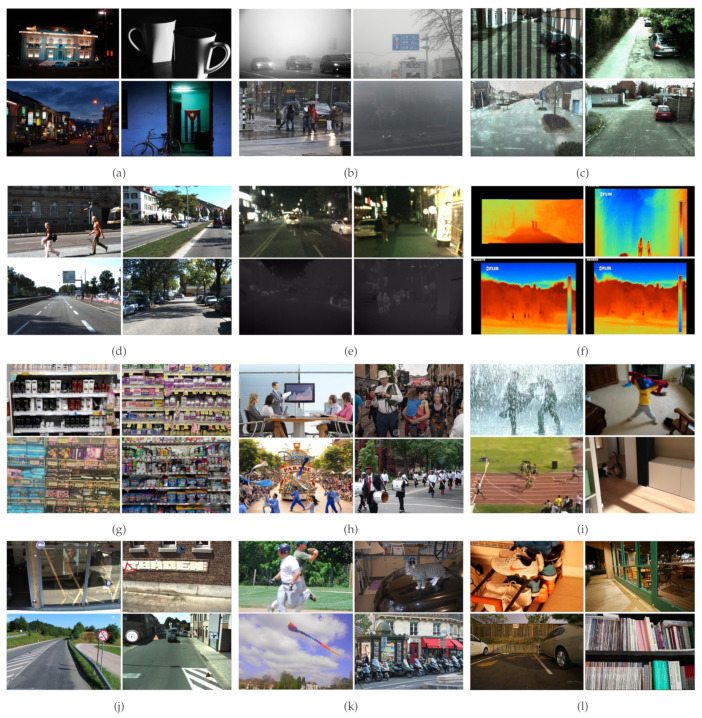
Sample images belonging to challenging datasets. (**a**) Represents samples taken from ExDark [97] showing low-light examples, (**b**) depicts samples from Reside [98] explaining harsh weather, (**c**) describes samples from CURE-TSD [92] illustrating example of camera distortion, lens flare, (**d**) denotes samples from KITTI [89] depicting various objects to be detected, (**e**) represents samples from Kaist [59] explaining saliency maps with their night-time images, (**f**) depicts samples taken from UNIRI-TID [55] showing example of thermal images, (**g**) highlights samples from SKU-110K [63] representing example of cluttered objects, (**h**) represents samples taken from Wider Face [72] showing faces at various angles, (**i**) represents samples taken from VOT-2018 [95] presenting example of complex indoor scenes, (**j**) shows samples taken from DFG [82] illustrating traffic signs at various places, (**k**) represents images taken from MS-COCO [12] describing example of objects in daily life and (**l**) outlines samples taken from see in the dark [53] dataset exhibiting examples captured at low illumination and high exposure.

One of the very few publicly available low-light datasets is the ExDARK (https://github.com/cs-chan/Exclusively-Dark-Image-Dataset accessed on 22 June 2021) dataset. Patil et al. [97] published this dataset in 2020. The dataset contains 7363 real-life low-light pictures from different low-light environments, such as indoors and at night-time. There are a total of 12 classes in the dataset. All the images in the dataset were manually collected, and afterwards, different image enhancement techniques such as de-hazing and blurring are applied to them. The classes of this dataset are table, cat, people, motorbike, dog, cup, chair, bicycle, boat, bottle, bus, car, and cat. As highlighted in Table 2, this dataset only contains information for the boundaries of objects. Figure 15a exhibits a few samples from this dataset. We have exploited this dataset to evaluate the performance of current state-of-the-art object detection algorithms on this dataset.

### 4.2. CURE-TSD

CURE-TSD [92] (https://github.com/olivesgatech/CURE-TSD accessed on 22 June 2021) is a large-scale publicly available dataset for the task of traffic sign detection. The dataset contains video sequences manually gathered by driving a car around different areas at different times of the day to capture diverse scenarios. Furthermore, the manually collected images are augmented to provide different kinds of occlusions such as rainy weather, decolorization, blur, darkening, dirty lens, exposure, codex error, snow, and haze. The authors of this dataset have suggested 34 videos for training, whereas 15 videos are allocated for testing purposes. Given five different levels of challenges to each type of occlusions, there is a total of 1.72 million frames. There are 14 types of traffic signs in this dataset labeled as speed limit, goods vehicles, no overtaking, no stopping, no parking, stop, bicycle, hump, no left, no right, priority to, no entry, yield, parking. Figure 15c illustrates few samples of this dataset. We have included this dataset in our benchmarking experiments.

### 4.3. RESIDE

RESIDE [98] is a publicly available dataset employed for the task of object detection in difficult weathers. The dataset contains multiple subdivisions, each for a separate task. However, we have worked on the Real-world Task-driven Testing Set (RTTS) subset. The subset RTTS contains 4332 real-world hazy images representing different scenarios in a day. Images were collected manually through video cameras and annotated with bounding boxes localizing objects. The dataset contains various real-world occlusions such as hazy, rainy, snowy weather, and so on. There are five classes annotated as bicycle, bus, motorbike, car, and person. Figure 15b depicts a few samples from this dataset. We incorporate this dataset along with ExDARK [97] and CURE-TSD [92] to benchmark the results on state-of-the-art object detection algorithms.

### 4.4. SKU-110K

SKU-100K (https://github.com/eg4000/SKU110K_CVPR19 accessed on 22 June 2021) is a new dataset collected by authors of the approach “Precise Detection in Densely Packed Scenes [63]” where they have explained that for challenging scenarios, overlapping detections can occur. The publishers of this dataset collected data from supermarkets and stores worldwide using cell cameras. The dataset only contains bounding box annotations for each image. The dataset contains 11,762 images. For training, the authors have suggested 8233 images, 588 images for validation, and 2941 images for testing. To ensure the same shop does not appear in more than one set, the authors recommended random selection of the sample images for training, validation, and testing. A few samples from the SKU-110K dataset are visible in Figure 15g.

### 4.5. UNIRI-TID

UNIRI-TID [55] (https://ieee-dataport.org/open-access/thermal-image-dataset-person-detection-uniri-tid accessed on 22 June 2021) is a novel dataset published in [54] for the task of detecting human/non-human objects in difficult weather. The dataset contains thermal images collected manually using the thermal camera in different weather conditions. The dataset was created to provide a balanced ratio of images for each kind of difficult weather such as rainy, fog, clear, and hazy. Furthermore, the creators of this dataset applied custom augmentation such as grayscale to increase the number of images. The dataset has a total of 35,974 images with 2663 images for clear weather, 2313 for rainy weather, 1135 for foggy weather, 18,333 are grayscale images, and the remaining 6111 images contain a mix of all possible weather conditions. Figure 15f illustrates a few samples of this dataset.

### 4.6. KAIST Multispectral Pedestrian Detection

KAIST Multispectral Pedestrian [59] (http://rcv.kaist.ac.kr/multispectral-pedestrian/ accessed on 22 June 2021) is a publicly available dataset containing both RGB and thermal images for the task of pedestrian detection. The dataset contains 95,000 images with thermal-RGB pairs captured by a camera mounted on a vehicle. The dataset contains challenging images captured during night-time and in harsh weather. Furthermore, the dataset provides scale, occlusions, and other challenges for object detection. The dataset only has two classes of pedestrians and background. A few samples of the dataset are illustrated in Figure 15e.

### 4.7. DFG Traffic Sign Dataset

DFG traffic sign dataset [82] (http://www.vicos.si/Downloads/DFGTSD accessed on 22 June 2021) is another dataset that provides ground truth for both traffic sign detection and recognition tasks. There is a total of seven thousand images collected with 200 different traffic signs. The complete dataset is divided into a training set with 5254 images and a test set with 1703 images. There is also an extended version of the dataset available with artificially augmented traffic signs. The extended version contains 15K traffic signs in natural images. Figure 15j exhibits a few examples of this dataset.

### 4.8. VT5000 Dataset

VT5000 dataset (https://github.com/lz118/RGBT-Salient-Object-Detection accessed on 22 June 2021) is the custom dataset curated by Tu et al. [69] for the task salient object detection. The dataset has 11 challenges collected in different scenes. There are 5500 pairs of RGB images with their corresponding thermal images. The main advantage of this dataset is that the sample images consist of objects with different sizes and varying illumination conditions.

### 4.9. Wider Face

Wider face dataset [72] (http://shuoyang1213.me/WIDERFACE/ accessed on 22 June 2021) is another dataset curated for detecting faces in complex scenarios. The dataset contains 32,203 images with high variance in scales, poses, and occlusions. Due to several tiny faces in the images, this dataset is one of the most challenging datasets for face detection. A few samples are visible in Figure 15h.

### 4.10. UFDD

UFDD [60] is a new face detection dataset that captures real issues not present in other datasets. The dataset contains images captured in different weather scenarios with rain, snow, and haze weather. Motion blur and focus blur have been manually added to images to increase the total number of images in the datasets. There is a total of 6452 images with 10,897 annotations.

### 4.11. See in the Dark

Another dataset that operates under low light is published by Chen et al. [53]. The dataset contains 5094 raw short-exposure images of both indoor and outdoor scenarios. Outdoor images are taken at night-time to add further complications. There are only bounding box annotations in the dataset. Therefore, it is an ideal dataset to employ object detection-based approaches. Figure 15l explains the dataset visually.

### 4.12. MS-COCO

Common Objects in Context (MS-COCO) (https://cocodataset.org/#download accessed on 22 June 2021) [12] is one of the biggest publicly available datasets introduced by Microsoft in 2015. The dataset contains 91 objects with 2.5 million labeled instances in 328,000 images. The dataset contains annotations for object detection tasks, instance segmentation, keypoints detection, panoptic segmentation, and salient object detection. The authors have split the dataset into three parts, with 82,783 images belonging to the training set, 40,504 images for the validation set, and 40,775 images for the test set. A few samples of the dataset are visible in Figure 15k.

### 4.13. VOT-2018

VOT-2018 [95] is a publicly available dataset for tracking challenges. The dataset has 60 sequences captured with the help of a camera. The data captured in the dataset present various challenges such as occlusion, illumination change, motion change, size change, and camera motion. The dataset facilitates the researchers to perform object detection and instance segmentation as well. A few instances from the dataset are shown in Figure 15i.

### 4.14. Kitti

KITTI [89] (http://www.cvlibs.net/datasets/kitti/index.php accessed on 22 June 2021) is one of the largest publicly available datasets. KITTI consists of several sub-datasets that perform various tasks such as image segmentation, 2D object detection, and 3D object detection. The data are collected with the help of LiDAR and various cameras mounted on vehicles and driving around the mid-size city of Karlsruhe, in rural areas, and on highways. More or less, each image has 20 annotated objects. There is a total of eight object classes. Figure 15d depicts few instances of this dataset.

## 5. Experiments

We have investigated the performance of current state-of-the-art object detection algorithms on the three most challenging datasets. The idea is to conduct an analysis that explains how well object detection algorithms can perform under harsh conditions. We employed Faster R-CNN [23], Mask R-CNN [25], YOLO V3 [33], Retina-Net [99], and Cascade Mask R-CNN [100] to benchmark their performance on the datasets of ExDARK [97], CURE-TSD [92], and RESIDE [98].

We have leveraged the capabilities of transfer learning in our experiments. All the object detection networks are incorporated with a backbone of ResNet50 [101] pre-trained on the COCO dataset [12]. We fine-tuned all the models for 15 epochs with a learning rate of $2\times 10^{-5}$ and used Adam [102] as an optimizer. We resized images to 800 × 800 during the training and testing phases.

## 6. Evaluation

This section discusses the well-known evaluation criteria essential to standardize state-of-the-art results for object detection in difficult situations. Moreover, this section analyzes the performance of the approaches discussed in Section 3 with quantitative and qualitative illustrations. Finally, we will present the outcome of our experiments on the three most widely exploited challenging datasets.

### 6.1. Evaluation Criteria

The standardization of how to assess the performance of approaches on unified datasets is imperative. Since object detection in a challenging environment is identical to generic object detection, the approaches appraise similar evaluation metrics.

#### 6.1.1. Precision

**Figure 16:**
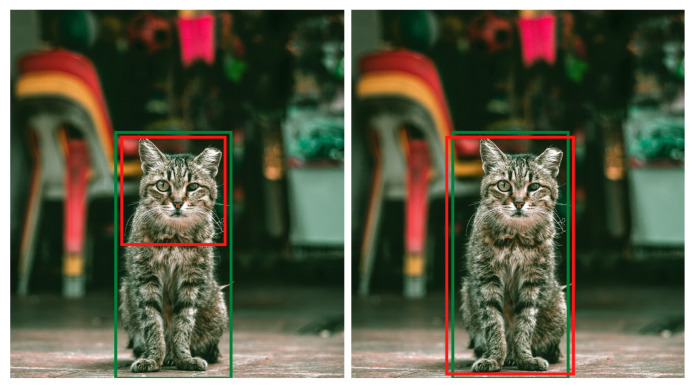
The image explains the visual difference between precise and imprecise prediction in object detection. The green color represents the ground truth, and the red color depicts the predicted boundary. Considering the IOU threshold value equals 0.5, the left prediction is not precise because the IOU between the ground truth and the inferred bounding box is less than 0.5. The bounding box prediction on the right side is precise because it covers almost the complete ground truth area.

Precision [103] defines as the percentage of a predicted region that belongs to the ground truth. Figure 16 illustrates an the difference between precise object detection and imprecise object detection. The formula for precision is explained below:

(1) $$\frac{\mathrm{Predicted}\;\mathrm{area}\;\mathrm{in}\;\mathrm{ground}\;\mathrm{truth}}{\mathrm{Total}\;\mathrm{area}\;\mathrm{of}\;\mathrm{predicted}\;\mathrm{region}}=\frac{\mathrm{TP}}{\mathrm{TP}\;+\;\mathrm{FP}}$$

where TP denotes true positives and FP represents false positives.

#### 6.1.2. Recall

Recall [103] is calculated as the percentage of the ground truth region that is present in the predicted region. The formula for the recall is given by:

(2) $$\frac{\mathrm{Ground}\;\mathrm{truth}\;\mathrm{area}\;\mathrm{in}\;\mathrm{predicted}\;\mathrm{region}}{\mathrm{Total}\;\mathrm{area}\;\mathrm{of}\;\mathrm{ground}\;\mathrm{truth}\;\mathrm{region}}=\frac{\mathrm{TP}}{\mathrm{TP}\;+\;\mathrm{FN}}$$

where TP is true positives and FN represents false negatives.

#### 6.1.3. F-Measure

F-measure [103] is computed by taking the harmonic mean of precision and recall. Mathematically, it is explained below:

(3) $$\frac{2\times \;\mathrm{Precision}\;\times \;\mathrm{Recall}}{\mathrm{Precision}\;+\;\mathrm{Recall}}$$

#### 6.1.4. Intersection Over Union

Intersection Over Union (IOU) [104] is one of the most important evaluation metrics that is regularly employed to determine the performance of object detection algorithms. It is the measure of how much the predicted region is overlapping with the actual ground truth region. IOU is defined as follows:

(4) $$\frac{\mathrm{Area}\;\mathrm{of}\;\mathrm{Overlap}\;\mathrm{region}}{\mathrm{Area}\;\mathrm{of}\;\mathrm{Union}\;\mathrm{region}}$$

#### 6.1.5. Average Precision (AP)

Average Precision computes average value of precision over different levels of recall. Higher the value of AP, the better performance and vice versa. The formula for calculating average precision is mentioned below:

(5) $$\mathrm{AP}=\sum_{n}\left(R_{n}-R_{n-1}\right)P_{n}$$

where *R_n_* and *P_n_* are the precision and recall at the *n*th threshold.

#### 6.1.6. Mean Absolute Error (MAE)

Mean absolute error measures average pixel-level absolute difference between predicted value and ground truth. It can be calculated as follows:

(6) $$\sum_{i=1}^{D}\left|x_{i}-y_{i}\right|$$

#### 6.1.7. Mean Average Precision

Mean Average Precision (mAP) is another extensively applied evaluation metric for category-specific object detection. The mAP is the mean of average precision computed over all the classes. Mathematically, it is explained by:

(7) $$\mathrm{mAP}=\frac{1}{N}\sum_{i=1}^{N}AP_{i}$$

where $AP_{i}$ is the average precision for a given class explained in Section 6.1.5 and *N* depics the total number of classes.

### 6.2. Evaluations for Object Detection in a Challenging Environment

Object detection in a challenging environment is used to distinguish objects from the background and regress their boundaries. Table 3 and Table 4 summarize the performance comparison of various methodologies that are explained in Section 3. Different approaches have reported results on specific evaluation metrics. Therefore, we have placed a “-” on the unused metric. By looking at Table 2, apart from [54,65], all the methods have reported results on separate datasets and utilized distinctive evaluation metrics. Therefore, the results summarized in these tables are not directly comparable to each other. Nonetheless, it is evident that there is still much room for improvement in all employed datasets. For instance, the method presented in [63] achieves the AP of 0.49 on SKU-110K dataset. Owing to the challenges present in the dataset (see Section 4.4), the method could only detect objects present in front of the camera or classify multiple objects with a single class. The approach introduced in [57] obtain the AP of 0.34 on the ExDark dataset, which contains dark indoor scenes make object detection even more challenging. Similarly, methods in [54,65] report mAPs of 0.35 and 0.68 on the KAIST dataset, which includes pair of thermal and RGB images taken at night-time or in harsh weather conditions (See Section 4.6).

Another technique that detects traffic signs on night-time images [62] reaches an mAP of 0.94 on the DFG dataset, which is elaborated on in Section 4.7. The method by Sasagawa et al. [48] detects objects with an mAP of 0.55 on the SID dataset that contains images captured under low light (see Section 4.11). Furthermore, Wang et al. [68] attain a mAP of 0.90 on the VOT-2018 dataset (see Section 4.13). The method introduced in [64] that converts night-time images to day time prior to object detection reaches a mAP of 0.82, and the approach from Kamal et al. [67] accomplish AP of 0.94 on the CURE-TSD dataset, which is explained in Section 4.2. For the sake of readability, we have presented the results of [61,72] in a separate table (Table 4) because they reported results on their own introduced evaluation metrics. It is critical to emphasize that, apart from the five methods [62,63,64,66,68], all other approaches have not discussed their computational performances.

**Figure 17:**
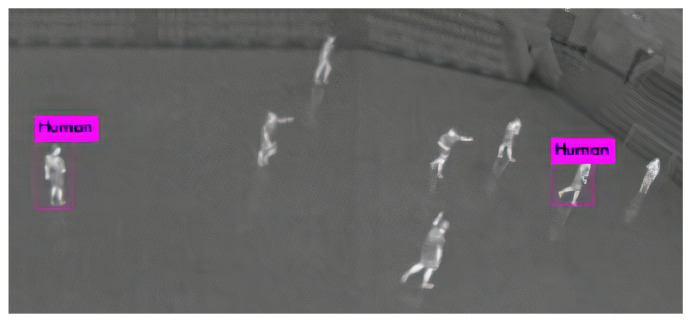
Failure case of the method proposed by Mate et al. [54]. Fails at detecting persons from the bird’s-eye view. Reprinted from [54].

**Figure 18:**
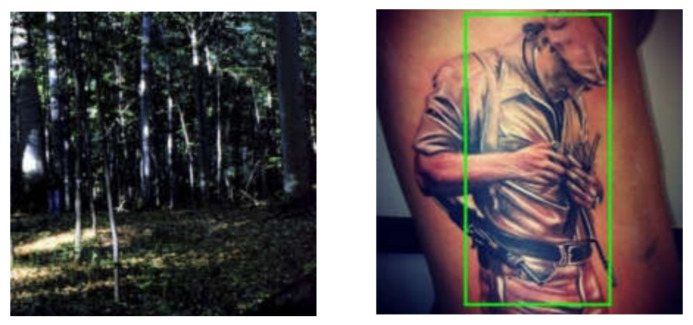
Failure case of the method proposed by Sarin et al. [58]. Human and face detector fails in low light and gets fooled by non-living human-like objects in images. Reprinted with the permission from [58]. Copyright 2015 IEEE.

Along with the quantitative analysis, we examined a few of the discussed approaches by paying attention to their failure cases. In Figure 17, we observed that from the bird’s-eye view, the method proposed by [54] fails to detect people. Similarly, the work of [58] gets confused with non-living human-like objects in images as exhibited in Figure 18.

**Figure 19:**
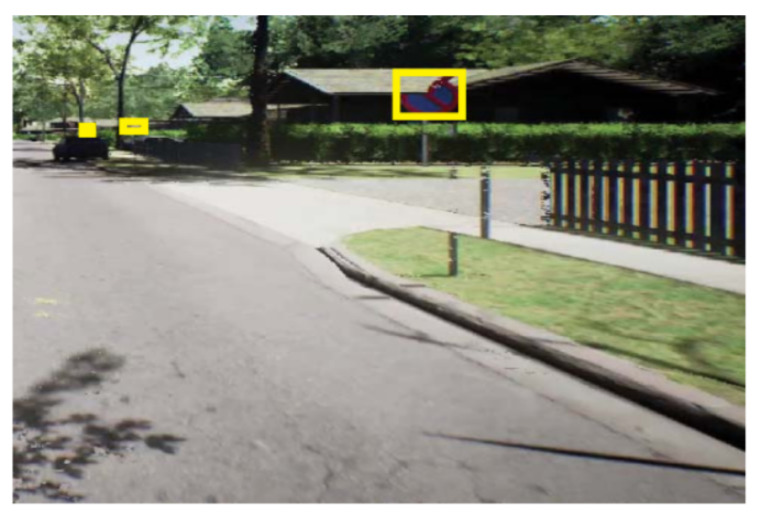
Failure case of the proposed method by Kamal et al. [67]. The proposed method fails if traffic signs are placed at different places. Adapted with the permission from [67]. Copyright 2019 IEEE.

**Figure 20:**
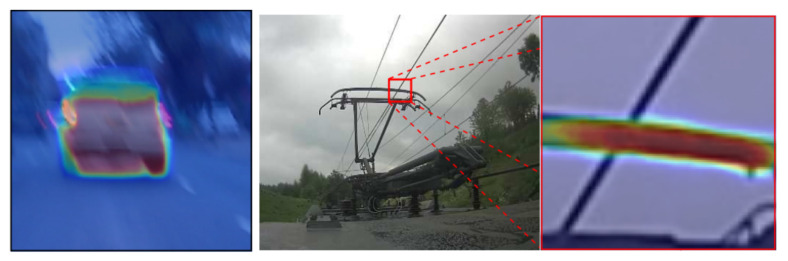
Failure case of the proposed method by Wang et al. [68]. The proposed method fails when faced with motion blur or a “non-object” pattern. Reprinted from [68].

The system of Kamal et al. [67] is vulnerable to detect traffic signs that are not close to the camera. A visual illustration is visible in Figure 19. Another approach presented by Wang et al. [68] is unable to detect objects in motion blur images. On top of that, the work tends to produce false positives on the images where no object is present. Figure 20 exhibits an example of such cases.

### 6.3. Evaluation of Our Experiments

The performance of current state-of-the-art object detection algorithms on challenging datasets is presented in Table 5. Since the task of object detection in challenging environments is analogous to a generic object detection problem, we have reported the results by employing the same evaluation metrics [12]. By looking at Table 5, it is evident that there is significant room for improvement on all of the employed datasets. For the ExDark dataset, YOLO V3 [33] produced the best results with an AP of 0.67. For CURE-TSD, Cascade Mask R-CNN [100] yields the best score, with an AP of 0.28. In the case of the RESIDE dataset, Mask R-CNN [25] shows the best results with an AP of 0.51. Furthermore, we investigate the computational capabilities of each employed object detection network on all three datasets in terms of frames per second. Owing to the lightweight one-stage architecture, YOLO V3 produces the best results with FPSs of 51, 50, and 50 on the datasets of ExDark, CURE-TSD, and RESIDE, respectively.

The main reason for the low performance of these state-of-the-art generic object detection algorithms is that they are not trained on challenging datasets that include low-light images or occluded images. Furthermore, the backbone network of these architectures cannot optimally extract the spatial features necessary for detecting objects in challenging environments. Hence, it is empirically established that generic object detection algorithms are not ideal for resolving object detection in challenging images.

## 7. Open Challenges and Future Directions

After reviewing many methodologies and benchmarking on object detection in a challenging environment, we have noticed several open issues that need to be highlighted to be tackled in future research.

**Standard evaluation criteria:** We observe that there has not been a defined standard while benchmarking on object detection in challenging situations. Some approaches have employed f1-score [54,69], whereas some have used mAP and AP to report their results [63,65,67]. Moreover, we notice that different IOU thresholds have been exploited to present the result, making the approaches incomparable to each other. Therefore, it is vital to establish a generic standard on which all future approaches working in this field can report the results.

**Real-time applications:** Similar to generic object detection, real-time challenging object detection is the growing need in practical scenarios. Generally, deep neural architectures require high computing power, which is unavailable in embedded devices. Therefore, it is essential to build methods that can optimally work on low computational resources. Furthermore, future research should discuss the computation of their proposed method, including the quantitative and qualitative analysis.

**Weak/unsupervised approaches:** To the best of our knowledge, all the current approaches tackling object detection in tricky environments with deep neural networks require large-scale annotated datasets. The creation of such datasets is an expensive and laborious process. Hence, there is a growing need to introduce challenging object detection systems that can train on no labels (unsupervised) or very few labels (weak supervised learning).

**Intelligent domain adaptation:** Current object detection systems under challenging environments work in similar types of challenges. For instance, a network trained on low-light images will not detect objects present in underwater images. However, we humans can adapt our learned knowledge into various domains. In the near future, we can expect a flurry of innovative ideas towards this promising direction.

**Leveraging GANs:** The deep neural network-based object detection systems demand enormous datasets for training networks. The ability of GANs to produce fake images can be highly beneficial to create large-scale challenging datasets. Moreover, most of the challenging datasets contain low-light, blurry, or not spatially rich images [59,97,98]. In future research, powers of recently published cycle GANs [105,106] could be exploited to transform images that are better suited for object detection algorithms.

**Combining spatial and textual information:** Modern object detection approaches under complex scenarios operate on CNN-based architectures that mainly depend on spatial features. However, images present in the challenging datasets are not spatially rich, resulting in suboptimal extracted features. Therefore, fusing textual information along with spatial information will facilitate the network to localize the objects. Nevertheless, developing efficient multimodal systems to detect objects in difficult conditions would be an exciting future direction.

## 8. Conclusions

With the rapid computational advances, noticeable improvements have been observed by employing deep learning-based object detection algorithms in challenging environments. This paper comprehensively reviews the relevant approaches that have attempted to identify objects under challenging scenarios through deep neural networks and provides an empirical performance analysis of currently employed object detection algorithms. Moreover, the advantages and limitations of all explained techniques are provided. Furthermore, this work describes all the relevant datasets currently exploited to resolve the problem of object detection under challenging environments. This work discusses the well-known evaluation criteria and presents a performance analysis of explained methodologies and object detection algorithms by benchmarking recent object detection algorithms on challenging datasets of ExDark [97], CURE-TSD [92], and Reside [98]. In the end, we highlight the current open issues and present future direction that can enhance the research of object detection under challenging environments.

## Figures and Tables

**Table 1:** A summary of advantages and limitations of methods tackling object detection in challenging environments. The double horizontal line separates the approaches according to the categories explained in Section 3.

Literature	Methods	Advantages	Limitations
Yuxan et al. [57]	Images are transformed and then fed into the RFB-Net (Section 3.1.2).	Context information fusion allows detection of object in low-light.	Relies on prior information about type of object, shape etc for detecting them in night-time.
Mate et al. [54]	Thermal images incorporated with YOLO (Section 3.1.1).	Thermal images give better information then RGB images in difficult conditions.	Fails at capturing from bird’s-eye view. Further, thermal images are not useful in environments where background and object temperature is same.
Ranjan et al. [61]	Single shot deep pyramid face detector (DPSSD).	Extract rich contextual information and multiscale features with help of pooling layers.	Requires a fixed input size.
Sasagawa et al. [48]	Fusion of pre-trained models using Glue layer and information distillation (Section 3.1.1).	Domain joining with help of glue layer reduces in computation and provides more information for models to learn from different domains.	Relies on prior domain knowledge.
Sarin et al. [58]	Single shot human and face detector (Section 3.1.3).	Simple and effective approach.	Fails in low light and gets fooled by human look alike pictures.
Avramovic et al. [62]	Region of interest(ROI) integration with Mask-RCNN and YOLO (Section 3.2.2).	Only choose regions of interest where objects of interest can occur instead of applying object detection to whole image.	Relies on prior knowledge of where objects can show.
Eran et al. [63]	Modification of CNN with Soft-IOU layer and custom EM-Merger layer.	Reduces overlapping detections (Section 3.2.3).	Treats overlapping predictions as clustering problem. Not very real run time.
Kun et al. [64]	Generative adversarial network with Faster R-CNN (Section 3.2.1).	Networks learns both day and night-time features.	Relies on prior information of converting night-time images to day time.
Ghose et al. [65]	Fusion of thermal images and their saliency maps using deep saliency networks (Section 3.3.1).	Provides rich contextual and depth information.	Relying on thermal images causes poor performance in day time or similar conditions.
Rashed et al. [66]	Fusion of RGB images with LiDAR sensors information with encoder–decoder architecture (Section 3.3.2).	Fusion of RGB, rgbFLow and lidarFlow provides greater information.	LiDAR sensors data and rgbFlow data need to be merged. There are multiple methods of fusion.
Kamal et al. [67]	Combination of SegNet and U-Net to detect traffic signs (Section 3.3.3).	Less computation cost as four corners of image are cropped and merged together before passing through network	Relies on prior information regarding where objects are most likely to occur. Fails when object location changes.
Wang et al. [68]	Fully convolutional Siamese networks with modified binary segmentation task (Section 3.3.4).	Pre-frame binary segmentation mask is used for low-level object representation instead of relying on feature extractor backbone.	Relies on prior information while generating binary segmentation mask. Fails when faced with motion blur and non-object pattern.
Zhengzheng et al. [69]	Two Stream convolutional neural network with attention mechanism (Section 3.4).	Fusion of rgb and thermal image to generate features and noise reduction with convolutional block attention module. A new dataset for benchmarking.	Thermal images are not efficient for every environment use.

**Table 2:** Important statistics of challenging object detection datasets. OD denotes object detection, SOD represents salient object detection, and COD is for category specific object detection.

Dataset	OD	SOD	COD	Samples	Type	Location
ExDark [97]	✓	✗	✓	7K	Camera Captured Image	https://github.com/cs-chan/Exclusively-Dark-Image-Dataset
CURE-TSD [92]	✓	✗	✓	1.72M	Camera Captured Videos	https://github.com/olivesgatech/CURE-TSD
UNIRI-TID [55]	✓	✗	✓	36K	Thermal Camera Captured Image	https://ieee-dataport.org/open-access/thermal-image-dataset-person-detection-uniri-tid
KAIST Multispectral Pedestrian Detection [59]	✓	✓	✗	95K	Camera Captured Image	https://soonminhwang.github.io/rgbt-ped-detection/
DFG Traffic Sign [82]	✓	✗	✓	7K	Camera Captured Image	http://www.vicos.si/Downloads/DFGTSD
VT5000 [69]	✓	✓	✗	5.5K	Camera Captured Image	https://github.com/lz118/RGBT-Salient-Object-Detection
Wider Face [72]	✓	✗	✗	32K	Camera Captured Image	http://shuoyang1213.me/WIDERFACE/
UFDD [60]	✓	✗	✗	6.4K	Camera Captured Image	https://ufdd.info/
See-in-the-Dark [53]	✓	✗	✓	5K	Camera Captured Raw	https://github.com/cchen156/Learning-to-See-in-the-Dark
MS-COCO [12]	✓	✓	✓	328K	Camera Captured Image	https://cocodataset.org/#download
VOT-2018 [95]	✓	✗	✗	60	Video captured sequences	https://www.votchallenge.net/vot2018/dataset.html
Kitti [89]	✓	✓	✓	1.5K	Camera Captured Image	http://www.cvlibs.net/datasets/kitti/index.php
SKU-110K [63]	✓	✗	✗	11.7K	Camera Captured Image	https://github.com/eg4000/SKU110K_CVPR19
Reside [98]	✓	✗	✓	4.3K	Camera Captured Image	https://github.com/Boyiliee/RESIDE-dataset-link

**Table 3:** Object detection performance analysis in challenging environments. IOU represents intersection over union threshold, whereas mAP and AP denote mean average precision and average precision, respectively. FPS represents frames per second calculated at inference time.

Literature	Year	Dataset	IOU	mAP	AP	F-Measure	Highlights	FPS
Eran et al. [63]	2019	SKU-110K	0.50:0.95	**-**	0.49	**-**	Deep IoU with EM-Merger (Section 3.2.3)	0.5
Yuxuan et al. [57]	2020	ExDark	0.50:0.95	**-**	0.34	**-**	RFB-Net (Section 3.1.2)	-
Zhengzheng et al. [69]	2020	VT5000	**-**	**-**	**-**	0.81	Attention-based SOD (Section 3.4)	-
Mate et al. [54]	2020	KAIST	0.50:0.95	0.35	**-**	0.36	YOLO (Section 3.1.1)	-
Ghose et al. [65]	2019	KAIST	0.50:0.95	0.68	**-**	**-**	Deep Saliency Networks (Section 3.3.1 )	-
Avramovic et al. [62]	2020	DFG	0.50	0.94	**-**	**-**	Mask R-CNN (Section 3.2.2)	2
Sasagawa et al. [48]	2020	SID	**-**	0.55	**-**	**-**	YOLO (Section 3.1.1)	-
Rashed et al. [66]	2019	KITTI	0.75	**-**	**-**	**-**	ShuffleSeg Segmentation Network (Section 3.3.2)	25
Wang et al. [68]	2019	VOT-2018	0.50	0.90	**-**	**-**	FC Siamese Networks (Section 3.3.4)	55
Kun et al. [64]	2019	Night-Dataset	**-**	0.82	**-**	**-**	GAN + Faster R-CNN (Section 3.2.1)	3.22
Kamal et al. [67]	2019	CURE-TSD	0.50:0.95	**-**	0.94	-	SegNet + U-Net (Section 3.3.3)	-

**Table 4:** Object detection performance: the results mentioned in this table are not directly comparable because different datasets and evaluation metrics have been used.

Literature	Year	Dataset	Evaluation Metric	Score	Highlights	FPS
Ranjan et al. [61]	2019	WIDER Face	True Accept Rate [61]	91.4%	Single Shot deep pyramid Face Detector	-
Sarin et al. [58]	2019	KAIST	True Positive Rate [58]	92.80%	Single Shot Human and Face Detector (Section 3.1.3)	-

**Table 5:** Object detection performance comparison on three challenging datasets. MR-CNN, FR-CNN, and CMR-CNN represent Mask R-CNN, Faster R-CNN, and Cascade Mask R-CNN, respectively. AP_s_ denotes average precision for small area at IOU = 0.50:0.95. AP_m_ represents average precision for medium area at IOU = 0.50:0.95 and AP_l_ depicts average precision for large area at IOU = 0.50:0.95. FPS highlights the speed of object detection networks at inference time in terms of frames per second.

Our Models	ExDark						CURE-TSD						RESIDE					
	AP	AP^50^	AP_s_	AP_m_	AP_l_	FPS	AP	AP^50^	AP_s_	AP_m_	AP_l_	FPS	AP	AP^50^	AP_s_	AP_m_	AP_l_	FPS
MR-CNN	0.54	0.84	0.22	0.46	0.59	8	0.20	0.35	0.2	0.17	0.37	5	**0.51**	**0.79**	**0.4**	0.11	**0.57**	8
FR-CNN	0.53	0.82	0.22	0.46	0.58	13	0.25	**0.43**	0.03	0.14	**0.41**	13	0.49	0.78	0.07	0.70	0.56	12
Yolo V3	**0.67**	**0.93**	**0.5**	**0.61**	**0.71**	51	0.16	0.32	0.05	0.03	0.26	50	0.37	0.78	0.07	**0.87**	0.56	50
Retina-Net	0.36	0.67	0.12	0.30	0.52	11	0.14	0.25	**0.12**	0.19	0.35	12	0.48	0.75	0.1	0.07	0.55	11
CMR-CNN	0.49	0.78	0.27	0.37	0.55	10	**0.28**	0.38	0.06	**0.23**	0.34	11	0.50	0.76	0.06	0.12	0.56	8

## Data Availability

Not applicable.
